# HIV-1 gp41-targeting fusion inhibitory peptides enhance the gp120-targeting protein-mediated inactivation of HIV-1 virions

**DOI:** 10.1038/emi.2017.46

**Published:** 2017-06-21

**Authors:** Qianqian Qi, Qian Wang, Weizao Chen, Lanying Du, Dimiter S Dimitrov, Lu Lu, Shibo Jiang

**Affiliations:** 1 Key Laboratory of Medical Molecular Virology of Ministries of Education and Health, Shanghai Medical College and Shanghai Public Health Clinical Center, Fudan University, Shanghai 200032, China; 2 Lindsley F. Kimball Research Institute, New York Blood Center, New York, NY 10065, USA; 3 Protein Interactions Section, Cancer and Inflammation Program, Center for Cancer Research, National Cancer Institute, National Institutes of Health, Frederick, MD 21702, USA

**Keywords:** entry inhibitor, gp120, gp41, HIV-1, viral inactivation

## Abstract

Protein- or peptide-based viral inactivators are being developed as novel antiviral drugs with improved efficacy, pharmacokinetics and toxicity profiles because they actively inactivate cell-free human immunodeficiency virus type 1 (HIV-1) virions before attachment to host cells. By contrast, most clinically used antiviral drugs must penetrate host cells to inhibit viral replication. In this study, we pre-treated HIV-1 particles with a gp120-targeting bispecific multivalent protein, 2Dm2m or 4Dm2m, in the presence or absence of the gp41-targeting HIV-1 fusion inhibitory peptides enfuvirtide (T20), T2635, or sifuvirtide (SFT). HIV-1 virions were separated from the inhibitors using PEG-6000, followed by testing of the residual infectivity of the HIV-1 virions. 2Dm2m and 4Dm2m exhibited significant inactivation activity against all HIV-1 strains tested with EC_50_ values at the low nanomolar level, whereas none of the gp41-targeting peptides showed inactivation activity at concentrations up to 250 nM. Notably, these three peptides significantly enhanced protein-mediated inactivation against cell-free HIV-1 virions, including HIV-1 laboratory-adapted and primary HIV-1 strains, as well as those resistant to T20 or T2635 and virions released from reactivated latently HIV-1-infected cells. These results indicate that the gp120-targeting bispecific multivalent proteins 2Dm2m and 4Dm2m have potential for further development as HIV-1 inactivator-based antiviral drugs for use in the clinic, either alone or in combination with a gp41-targeting HIV-1 fusion inhibitor such as T20, to treat patients with HIV-1 infection and AIDS.

## INTRODUCTION

Entry of human immunodeficiency virus type 1 (HIV-1) into the target cell is initiated by binding of gp120, the surface subunit of HIV-1 envelope glycoprotein (Env), to the receptor CD4 and co-receptor CXCR4 or CCR5 on the target cell.^1, 2^ This event triggers a cascade of conformational changes in gp41 from the native, pre-fusion form of Env to a highly stable post-fusion structure, a hairpin-like six-helix bundle (6-HB) formed between three molecules of the N-terminal heptad repeat (NHR) and the C-terminal heptad repeat (CHR) of gp41. Subsequently, the HIV-1 virion fuses with the cellular membrane, and the viral RNA enters the target cell.^3, 4^ Therefore, both gp120 and gp41 are important targets for the development of HIV-1 entry inhibitors or viral inactivators, which are expected to inactivate virions before attachment to the host cells.^5, 6^

The soluble form of human CD4 (sCD4) is a potential HIV-1 inactivator because it can induce the inactivation of HIV-1 virions by targeting the CD4-binding site (CD4bs) on gp120. However, the viral inactivation activity of sCD4 is dose- and temperature-dependent because of the reversible blockage of receptor binding.^7^ In addition, at low concentrations, sCD4 actually increases HIV-1 infectivity in CD4^−^CCR5^+^ cells.^8^ D1D2, the first two domains of CD4, were subsequently investigated as an anti-HIV-1 drug candidate. The HIV-1 inhibitory activity of D1D2 is high,^9^ but its stability is low, and it binds to CD4^+^ T cells and human B cells in the absence of HIV-1.^10^ To overcome these disadvantages, we developed mD1.22, which comprises the first single domain of D1D2 and is stable in isolation and highly soluble. It exhibits high expression, stability, ligand specificity and affinity, as well as potent and broad HIV-1 inhibitory activity.^10^ However, mD1.22 targets only CD4bs on gp120 and may not be highly effective against HIV-1 with mutations at this site.

**Figure 1:**
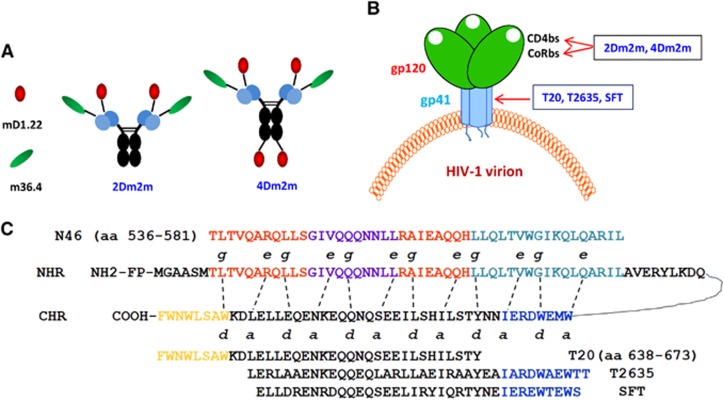
Anti-HIV-1 molecules tested in the present study. (**A**) Schematic view of the gp120-targeting proteins 2Dm2m and 4Dm2m. (**B**) The targeting sites of the HIV-1 attachment inhibitors (2Dm2m and 4Dm2m) and the HIV fusion inhibitors (T20, T2635 and SFT). The CD4-binding site, CD4bs; the co-receptor binding site, CoRbs; an engineered single human CD4 domain targeting CD4bs in gp120, mD1.22; a potent neutralizing monoclonal antibody targeting CoRbs in gp120, m36.4; sifuvirtide, SFT. (**C**) Schematic view of the HIV-1 gp41 molecule and interactions between the CHR and NHR domains, as well as the CHR-derived fusion inhibitory peptides.

The co-receptor binding site (CoRbs), also known as CD4-induced site (CD4i), is the most conserved region on gp120.^11, 12^ We identified a human domain antibody (dAb) targeting CoRbs, m36 and its variant m36.4 with highly potent HIV-1 neutralizing activity.^13, 14^ We subsequently designed and engineered two bispecific multivalent proteins, 2Dm2m and 4Dm2m, containing 2 and 4 copies of mD1.22, respectively, and 2 copies of m36.4 (Figure 1A), which are expected to target both CD4bs and CoRbs on gp120 (Figure 1B). These bispecific multivalent proteins have potent inhibitory activity against a broad spectrum of HIV-1 strains and high stability, with great potential to be further developed as novel anti-HIV therapeutics.^15^

In this study, we aimed to investigate whether 2Dm2m and 4Dm2m can inactivate cell-free HIV-1 particles when used alone or in combination with a gp41-targeting peptide, such as T20,^16^ T2635,^17^ or SFT^18^ (Figure 1C). The outcome of this study is expected to have implications for the rational design of an efficacious HIV-1 therapeutic strategy for the inactivation of cell-free virions and inhibition of viral–cellular membrane fusion, as well as the treatment of HIV-1/AIDS patients who fail to respond to current antiretroviral therapy.

## MATERIALS AND METHODS

### Peptides, cells and virus

The peptides T20, T2635 and SFT were synthesized by a standard solid-phase fluorenylmethoxycarbonyl method and had a purity of >95%. The concentrations of these peptides were measured according to Edelhoch’s method.^19^ MT-2, TZM-b1 and ACH-2 cells, HIV-1 laboratory-adapted strains, primary HIV-1 isolates and T20-resistant strains were obtained from the National Institutes of Health AIDS Reagent Program. T2635-resistant HIV-1 strains were kindly provided by Dr Rogier W. Sanders.^20, 21^

### Expression of proteins

The two bispecific multivalent proteins, 2Dm2m and 4Dm2m, were expressed by 293 FreeStyle cells as described previously.^13^ Briefly, the positive plasmid was transformed into prepared 293 FreeStyle cells by calcium phosphate, and the medium was replaced with fresh medium after incubation for 6 h. The culture supernatant was collected after 3 days of incubation, followed by the purification of proteins using Protein A Sepharose 4 Fast Flow (GE Healthcare, Piscataway, NJ, USA) column chromatography.

### Detection of inactivation activity against cell-free HIV-1 virions

The inactivation activity of the proteins and peptides alone or in combination against cell-free HIV-1 virions, including HIV-1 laboratory-adapted strains, primary HIV-1 isolates, and T20- and T2635-resistant strains, was tested as previously described.^22^ In brief, 100 μL of protein (starting concentration: 100 nM) or peptide (starting concentration: 250 nM) at a series of dilutions was incubated with 100 μL of virus at 500 TCID_50_/mL at 4 °C for 1 h before the addition of PEG-6000 at a final concentration of 3%. After incubation for another 1 h at 4 °C, the mixture was centrifuged at 15 000 r/min at 4 °C for 30 min. The supernatant was removed, and the pellet was washed with 3% PEG-6000 in PBS containing 10 mg/mL BSA three times to separate the HIV-1 particles from protein or peptide. Then, the viral pellet was resuspended in 100 μL of culture medium, followed by the addition of 100 μL of MT-2 or TZM-b1 cells (1 × 10^5^/mL). After 72 h of infection, the culture medium was removed, and the cells were washed with PBS, followed by the addition of a lysing reagent and measurement of luciferase activity using a luciferase kit (Promega, Madison, WI, USA) with a luminometer (Infinite F200pro, Tecan, Männedorf, Switzerland). At 4 days post infection, 100 μL of the MT-2 cell culture supernatant was collected and mixed with an equal volume of 5% Triton X-100 to measure p24 antigen by ELISA as previously described.^23^ The EC_50_ (effective concentration causing 50% inactivation) was calculated using the CalcuSyn program, as described previously.^24^ The fold enhancement of the inhibitor’s potency in combination was calculated by dividing the EC_50_ value of the inhibitor alone by the EC_50_ value of the inhibitor when combined with another inhibitor.

### Detection of inactivation activity against released HIV-1 virions

The inactivation activity of the proteins or peptides either alone or in combination against HIV-1 virions released from reactivated latently HIV-1-infected cells was detected. Briefly, the latently infected ACH-2 cells were incubated with romidepsin,^25^ also known as Istodax, for three days before collecting the culture supernatants containing released HIV-1 particles. Then, 100 μL of the collected culture supernatant was incubated with 100 μL of protein (starting concentration: 100 nM) or peptide (starting concentration: 250 nM) at a series of dilutions at 4 °C for 1 h before the addition of PEG-6000 at a final concentration of 3%. After incubation at 4 °C for 1 h, the mixture was centrifuged at 15 000 r/min at 4 °C for 30 min. The supernatant was removed, and the pellet was washed with 3% PEG-6000 in PBS containing 10 mg/mL BSA three times to separate the HIV-1 pellet from the protein or peptide. The viral pellet was then resuspended in 100 μL of culture medium, followed by measurement of the residual infectivity of the virions and calculation of the EC_50_ and fold enhancement as described above.

## RESULTS

### The gp120-targeting proteins sCD4, D1D2, mD1.22, m36.4, 2Dm2m and 4Dm2m could inactivate HIV-1 virions with different potencies

**Figure 2:**
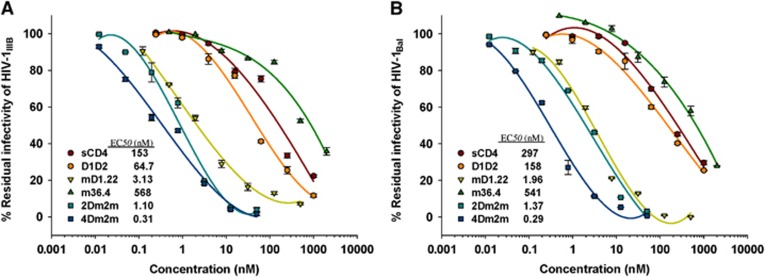
Inactivation of HIV-1 laboratory-adapted strains by the gp120-targeting proteins. (**A**) Inactivation of HIV-1_IIIB_ virions by sCD4, D1D2, mD1.22, m36.4, 2Dm2m and 4Dm2m. (**B**) Inactivation of HIV-1_Bal_ virions by sCD4, D1D2, mD1.22, m36.4, 2Dm2m and 4Dm2m.

We first investigated the inactivation potency of the gp120-targeting proteins, including sCD4, D1D2, mD1.22, m36.4, 2Dm2m and 4Dm2m, against the HIV-1 laboratory-adapted strains IIIB (Subtype B, X4) and Bal (Subtype B, R5). As shown in Figure 2, the bispecific multivalent proteins 2Dm2m and 4Dm2m, which target CD4bs and CoRbs on gp120, displayed high efficiency for inactivation of cell-free HIV-1 virions, with EC_50_ values of ~1 and ~0.3 nM, respectively. However, the monovalent and monospecific proteins sCD4, D1D2 and mD1.22, which target CD4bs on gp120, and m36.4, which targets CoRbs on gp120, showed significantly lower efficiency for the inactivation of cell-free HIV-1 virions, with EC_50_ values of 153–297, 65–158, 2.0–3.1 and 541–568 nM, respectively. These results suggest that the gp120-targeting multivalent bispecific proteins 2Dm2m and 4Dm2m are more effective than the gp120-targeting monovalent and monospecific proteins in inactivating cell-free HIV-1 virions.

### The gp120-targeting multivalent bispecific proteins exhibit potent viral inactivation activity against a broad spectrum of HIV-1 strains, whereas the gp41-targeting fusion inhibitory peptides have no viral inactivation activity

Next, we tested the inactivation activity of the bispecific proteins targeting gp120, that is, 2Dm2m and 4Dm2m, and the fusion inhibitory peptides targeting gp41, that is, T20, T2635 and SFT, against the laboratory-adapted HIV-1 strains, that is, IIIB and Bal, and primary HIV-1 isolates with different subtypes and tropisms, including US4 (GS007) (Subtype B, R5), 92UG024 (Subtype D, X4), 92TH009 (Subtype A/E, R5) and BCF02 (Subtype O, R5). As shown in Table 1, the EC_50_ values of 2Dm2m and 4Dm2m for inactivating HIV-1 laboratory-adapted virions were ~1 and ~0.3 nM, respectively, whereas T20, T2635 and SFT exhibited no HIV-1-inactiviating activity at concentrations up to 250 nM. Similarly, the EC_50_ values of 2Dm2m and 4Dm2m for inactivating HIV-1 primary isolates were 0.9–11 and 0.2–1.9 nM, respectively (Table 1), whereas the gp41-targeting peptides showed no inactivation activity against these HIV-1 primary isolates.

T20 can easily induce HIV-1 mutants resistant to T20 in HIV-1/AIDS patients treated with T20,^26, 27^ and HIV-1 variants with mutations in the HIV-1 gp41 NHR region are also resistant to T2635 and SFT.^20, 28^ Here we measured the inactivation activity of the proteins 2Dm2m and 4Dm2m and the peptides T20, T2635 and SFT against T20- and T2635-resistant HIV-1 strains. As shown in Table 1, both the gp120-targeting proteins 2Dm2m and 4Dm2m effectively inactivated T20- and T2635-sensitive or T20- and T2635-resistant HIV-1 strains, with EC_50_ values of 1.7–5.5 and 0.8–3.4 nM, respectively, whereas none of the three gp41-targeting peptides showed significant viral inactivation activity. These results suggest that in contrast to the gp41-targeting peptides, the gp120-targeting proteins 2Dm2m and 4Dm2m are effective in inactivating cell-free HIV-1 virions, including those resistant to gp41-targeting peptides.

### The gp41-targeting peptides enhance the protein-mediated inactivation of laboratory-adapted and primary HIV-1 strains

We then tested the inactivation activity of the gp120-targeting multivalent bispecific proteins against cell-free HIV-1 virions, including the HIV-1 laboratory-adapted strains and primary HIV-1 isolates, in the presence or absence of the gp41-targeting peptides T20, T2635, and SFT. As shown in Table 2, 2Dm2m and 4Dm2m showed potent inactivation activity against cell-free HIV-1_IIIB_ and HIV-1_Bal_ virions, with EC_50_ values of ~2 and ~0.3 nM, respectively. In the presence of the peptide T20, T2635 or SFT, 2Dm2m- and 4Dm2m-mediated inactivation activities against HIV-1_IIIB_ and HIV-1_Bal_ particles were increased by 1.4- to 5.4-fold and 1.6- to 2.5-fold, respectively.

In the absence of the gp41-targeting peptides, 2Dm2m and 4Dm2m effectively inactivated the cell-free virions of the primary HIV-1 isolates US4(GS007), 92UG024, 92TH009 and BCF02 with EC_50_ values of 0.9–11 and 0.2–1.9 nM, respectively. In the presence of the gp41-targeting peptides T20, T2635 or SFT, 2Dm2m- and 4Dm2m-mediated inactivation activity against these primary HIV-1 virions was increased by 1.8- to 63-fold and 1.2- to 10-fold, respectively (Table 3). These results suggest that the gp41-targeting peptide-based anti-HIV-1 drug T20 or next-generation fusion inhibitor T2635 or SFT can be used in combination with the gp120-targeting proteins 2Dm2m and 4Dm2m to enhance protein-mediated inactivation against cell-free HIV-1 virions, including laboratory-adapted strains and primary HIV-1 isolates.

### The gp41-targeting peptides enhance the protein-mediated inactivation of T20- and T2635-resistant HIV-1 strains

We subsequently assessed the protein-mediated inactivation of T20- and T2635-resistant HIV-1 virions in the presence or absence of the gp41-targeting fusion inhibitory peptides T20, T2635 and SFT. In the absence of the gp41-targeting fusion inhibitory peptide T20, 2Dm2m and 4Dm2m effectively inactivated the T20-resistant strain (9491) with EC_50_ values of 5.8 and 3.7 nM, respectively. In the presence of T20, 2Dm2m/4Dm2m-mediated inactivation of the T20-resistant strain was increased by ~5.5-fold and 4.2-fold, respectively (Table 4). Similarly, 2Dm2m or 4Dm2m alone was effective in inactivating the T2635-resistant strain (K90E/N126K), with EC_50_ values of 3.6 and 2.2 nM, respectively, while in the presence of the peptide T20, T2635 or SFT, 2Dm2m/4Dm2m-mediated inactivation against the T2635-resistant strain was increased by approximately 3.3- to 3.9-fold and 4.5- to 6.5-fold, respectively (Table 4). These results suggest that the gp41-targeting peptides can significantly enhance the inactivation activity of 2Dm2m or 4Dm2m against T20- and T2635-resistant strains.

### The gp41-targeting peptides enhance the gp120-targeting protein-mediated inactivation of reactivated HIV-1 virions from ACH-2 cells

Finally, we measured the inactivation activity of 2Dm2m and 4Dm2m against HIV-1 virions released from latently HIV-1-infected ACH-2 cells reactivated with romidepsin, a histone deacetylase inhibitor (HDACi),^25^ in the presence or absence of the gp41-targeting peptide T20, T2635 or SFT. As shown in Table 5, 2Dm2m or 4Dm2m alone effectively inactivated the released HIV-1 virions, with EC_50_ values of ~1.6 and ~0.7 nM, respectively. In the presence of the gp41-targeting peptide T20, T2635 or SFT, the inactivation activity of 2Dm2m and 4Dm2m was enhanced by 3.2- to 5.9-fold and 4.4- to 6.0-fold, respectively, suggesting that reactivated HIV-1 virions from latently infected cells can be inactivated by the multivalent bispecific proteins 2Dm2m and 4Dm2m and that their inactivation activity can be enhanced by fusion inhibitory peptides targeting the gp41 NHR domain.

## DISCUSSION

Thirty-two individual anti-HIV drugs, including nucleoside reverse-transcriptase inhibitors, such as epivir, emtriva and retrovir; nonnucleoside reverse-transcriptase, including rescriptor, sustiva, intelence and viramune; and protease inhibitors, such as agenerase, aptivus and saquinavir; the integrase inhibitor raltegravir; and entry inhibitors, including enfuvirtide (T20) and maraviroc (UK-427,857), have been approved by the United States Food and Drug Administration (FDA) for treatment of HIV infection/AIDS (http://www.fda.gov/ForPatients/Illness/HIVAIDS/Treatment/ucm118915.htm). However, these antiretroviral drugs must permeate the cell or attach to the host cell surface to inhibit viral replication or block viral fusion, and none can actively attack and inactivate cell-free virions in the blood circulation before attachment to the target cells. Soluble CD4 (sCD4), which comprises the four extracellular domains (D1 to D4) of human CD4, can inactivate cell-free HIV-1 particles by blocking the attachment of viral gp120 to host cells. However, its low binding affinity to gp120 and potential to increase HIV-1 infection in CD4^−^CCR5^+^ cells have prevented its further development.^29^ To improve the neutralizing activity of sCD4, several groups have developed fusion proteins by linking CD4 or CD4 domain(s) to human IgG or a monoclonal antibody, such as CD4-Ig,^8^ CD4-IgG2 (PRO 542)^30^ and sCD4-17b.^31, 32^ However, CD4-Ig can enhance HIV-1 infection in the presence of co-receptor,^33^ whereas CD4-IgG2 is less effective against infection by X4 viruses, and large doses are required to reduce HIV-1 replication in patients.^34^ The neutralization breadth of sCD4-17b is limited.^31^

The engineered multivalent bispecific proteins 2Dm2m and 4Dm2m are superior to the fusion proteins described above in terms of their anti-HIV-1 potency, breadth, stability and solubility.^15, 35^ In this study, all HIV-1 strains tested were sensitive to the inactivating activity of the bispecific proteins 2Dm2m and 4Dm2m. For example, 4Dm2m inactivated HIV-1 virions, including laboratory-adapted HIV-1 strains, primary HIV-1 isolates and T20-/T2635-resistant strains, as well as virions released from reactivated latently infected cells, with EC_50_ at the low nanomolar level. However, the HIV-1 fusion inhibitory peptides T20, T2635 and SFT showed no significant inactivation activity against cell-free HIV-1 virions. Interestingly, these gp41-targeting HIV-1 fusion inhibitory peptides effectively enhanced the gp120-targeting protein-mediated inactivation of HIV-1 virions of divergent HIV-1 strains, including those resistant to T20 and T2635. Our results demonstrate that the gp120-targeting bispecific proteins can be developed as effective HIV-1 inactivators and that these gp41-targeting HIV-1 fusion inhibitory peptides can be used in combination with 2Dm2m or 4Dm2m to enhance their viral inactivation activity.

**Figure 3:**
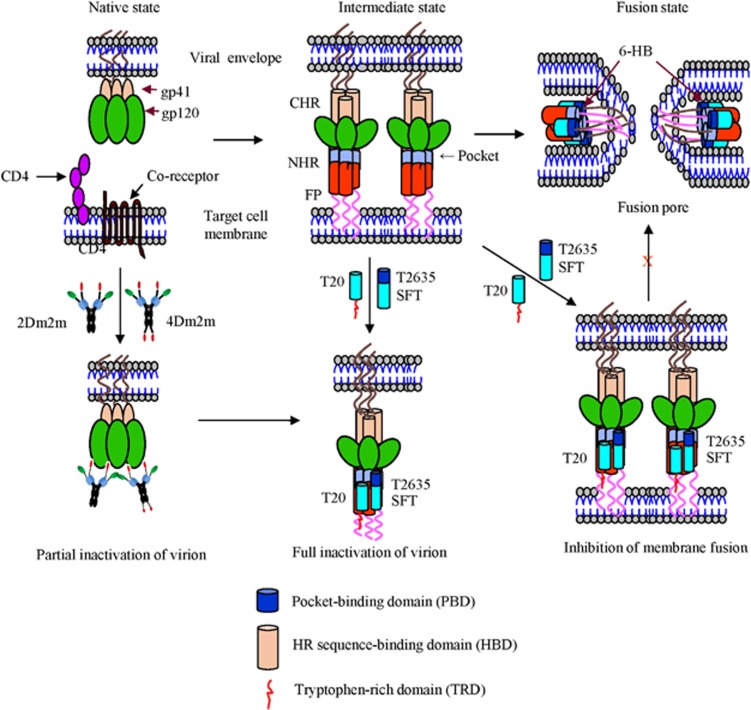
Putative mechanisms of action of bispecific proteins and/or HIV-1 fusion inhibitory peptides to inactivate virions and inhibit viral fusion and entry. 2Dm2m or 4Dm2m binds to gp120 via its mD1.22 and m36.4 domains, resulting in the partial inactivation of the virion. Binding of 2Dm2m or 4Dm2m to gp120 induces the exposure of the gp41 trimer, to which the HIV-1 fusion inhibitory peptide, T20, T2635 or SFT, binds, leading to the full inactivation of the virion. The T20, T2635 or SFT peptide can also bind to the exposed gp41 trimer induced by CD4 on the target cell, causing inhibition of viral–cell membrane fusion. Sifuvirtide, SFT.

What is the mechanism by which the gp120-targeting proteins 2Dm2m and 4Dm2m inactivate cell-free HIV-1 virions, and why do gp41-targeting peptides enhance the gp120-targeting protein-mediated inactivation of HIV-1 virions? HIV-1 infection begins with binding of gp120, through CD4bs and CoRbs, to the primary receptor CD4 and the co-receptor, CCR5 or CXCR4, on the target cell, triggering the formation of the 6-HB fusion core structure by CHR and NHR in gp41.^36^ Like sCD4 and mD1.22, 2Dm2m and 4Dm2m can bind to the CD4bs on gp120 to trigger the premature conformational change of gp120, resulting in the exposure of CoRbs on gp120, which can be bound by the m36.4 domain in 2Dm2m and 4Dm2m. Therefore, the HIV-1 virion is partially inactivated and is unable to bind the primary receptor, CD4, and the co-receptor, CCR5 or CXCR4. Subsequently, the 2Dm2m- or 4Dm2m-bound gp120/gp41 complex further changes conformation to expose the gp41 trimer. Binding of the gp41-targeting fusion inhibitory peptide (for example, T20, T2635 or SFT) to the exposed gp41 trimer enhances gp120-targeting protein-mediated virion inactivation (Figure 3). In addition, the gp41 CHR-derived peptides, T20, T2635 and SFT, can independently bind to the NHR of viral gp41 to prevent viral-cell membrane fusion by interrupting the formation of 6-HB (Figure 3), leading to the inhibition of HIV-1 fusion with the target cells.^4, 5^ Therefore, the combined use of a gp41-targeting protein, that is, 2Dm2m or 4Dm2m, and a gp41-targeting peptide such as T20 can have dual effects to inactivate cell-free virions and to inhibit virus fusion with the target cells.

In summary, this study has demonstrated that the multivalent bispecific proteins 2Dm2m and 4Dm2m, which target both CD4bs and CoRbs in gp120, can effectively inactivate cell-free HIV-1 virions before attachment to the receptor CD4 and co-receptor, CCR5 or CXCR4, on the target cells. Although gp41-targeting peptides, including T20, SFT and T2635, cannot inactivate HIV-1 virions, they can enhance the inactivation activity of 2Dm2m or 4Dm2m when one of the peptides is used in combination with the protein. These findings suggest that the multivalent bispecific proteins 2Dm2m and 4Dm2m have good potential to be further developed as HIV-inactivator-based antiviral therapeutics to treat HIV-1/AIDS patients, either alone or in combination with a gp41-targeting peptide or other antiretroviral drugs.

## Figures and Tables

**Table 1:** Inactivation of 2Dm2m, 4Dm2m, T20, T2635 and SFT against laboratory-adapted HIV-1 strains, primary HIV-1 isolates, and T20-/T2635-sensitive and T20-/T2635-resistant HIV-1 strains

**Viruses**	**EC_50_ (nM)**				
	**2Dm2m**	**4Dm2m**	**T20**	**T2635**	**SFT**
*Laboratory-adapted HIV-1 strains*					
IIIB (B, X4)	0.98±0.15	0.28±0.01	>250	>250	>250
Bal (B, R5)	1.37±0.06	0.29±0.02	>250	>250	>250

*Primary HIV-1 isolates*					
US4 (GS007) (B, R5)	0.88±0.01	0.15±0.01	>250	>250	>250
92UG024 (D, X4)	11.0±0.70	1.77±0.16	>250	>250	>250
92TH009 (A/E, R5)	1.78±0.07	0.39±0.04	>250	>250	>250
BCF02 (O, R5)	2.86±0.76	1.91±0.46	>250	>250	>250

*T20-sensitive and T20-resistant HIV-1 strains*					
9489	1.73±0.20	0.78±0.06	>250	>250	>250
9491	5.47±0.92	3.37±0.34	>250	>250	>250

*T2635-sensitive and T2635-resistant HIV-1 strains*					
WT	2.74±0.30	1.09±0.02	>250	>250	>250
K90E/N126K	3.51±0.09	1.93±0.05	>250	>250	>250

Abbreviations: T20-sensitive strain, 9489; T20-resistant strain, 9491; T2635-sensitive strain, WT; T2635-resistant strain, K90E/N126K.

Data are representative of testing in triplicate (mean±sd).

**Table 2:** Enhancement of the viral inactivation activity of the gp120-targeting proteins against laboratory-adapted HIV-1 virions in the absence (alone) or presence (in mixture) of gp41-targeting fusion inhibitor T20, T2635 or SFT

**Combined with**	**2Dm2m**			**4Dm2m**		
	**EC_50_ (nM)**		**Enhancement (fold)**	**EC_50_ (nM)**		**Enhancement (fold)**
	**Alone**	**In mixture**		**Alone**	**In mixture**	
*HIV-1_IIIB_ (B, X4)*						
T20	0.99	0.23	4.3	0.39	0.06	6.5
T2635	0.99	0.21	4.7	0.39	0.10	3.9
SFT	0.99	0.61	1.6	0.39	0.15	2.6

*HIV-1_Bal_ (B, R5)*						
T20	1.29	0.24	5.4	0.27	0.11	2.5
T2635	1.29	0.34	3.8	0.27	0.11	2.5
SFT	1.29	0.93	1.4	0.27	0.17	1.6

Data are representative of testing in triplicate (mean).

**Table 3:** Enhancement of viral inactivation activity of the gp120-targeting proteins against primary HIV-1 virions in the absence (alone) or presence (in mixture) of the gp41-targeting fusion inhibitor T20, T2635 or SFT

**Combined with**	**2Dm2m**			**4Dm2m**		
	**EC_50_ (nM)**		**Enhancement (fold)**	**EC_50_ (nM)**		**Enhancement (fold)**
	**Alone**	**In mixture**		**Alone**	**In mixture**	
*US4(GS007) (B, R5)*						
T20	0.88	0.49	1.8	0.16	0.13	1.2
T2635	0.88	0.17	5.2	0.16	0.08	2.0
SFT	0.88	0.27	3.3	0.16	0.10	1.6

*92UG024 (D, X4)*						
T20	11.4	2.38	4.8	1.85	0.25	7.4
T2635	11.4	0.18	63	1.85	0.18	10
SFT	11.4	0.24	47	1.85	0.27	6.9

*92TH009 (A/E, R5)*						
T20	1.76	0.22	8.0	0.39	0.18	2.2
T2635	1.76	0.27	6.5	0.39	0.12	3.3
SFT	1.76	0.28	6.3	0.39	0.17	2.3

*BCF02 (O, R5)*						
T20	3.79	1.69	2.2	1.77	1.12	1.6
T2635	3.79	0.95	4.0	1.77	0.30	5.9
SFT	3.79	0.90	4.2	1.77	1.36	1.3

Data are representative of testing in triplicate (mean).

**Table 4:** Enhancement of viral inactivation activity of the gp120-targeting proteins against T20-/T2635-resistant HIV-1 virions in the absence (alone) or presence (in mixture) of the gp41-targeting fusion inhibitor T20, T2635 or SFT.

**Combined with**	**2Dm2m**			**4Dm2m**		
	**EC_50_ (nM)**		**Enhancement (fold)**	**EC_50_ (nM)**		**Enhancement (fold)**
	**Alone**	**In mixture**		**Alone**	**In mixture**	
*T20-resistant HIV-1 strain (9491)*						
T20	5.83	1.06	5.5	3.69	0.89	4.2

*T2635-resistant HIV-1 strain (K90E/N126K)*						
T20	3.61	0.92	3.9	2.14	0.36	5.9
T2635	3.61	1.24	2.9	2.14	0.33	6.5
SFT	3.61	1.09	3.3	2.14	0.48	4.5

Data are representative of testing in triplicate (mean).

**Table 5:** Enhancement of the inactivation activity of the gp120-targeting proteins against released virions from reactivated HIV-1 latent cells in the absence (alone) or presence (in mixture) of the gp41-targeting fusion inhibitor T20, T2635 or SFT

**Combined with**	**2Dm2m**			**4Dm2m**		
	**EC_50_ (nM)**		**Enhancement (fold)**	**EC_50_ (nM)**		**Enhancement (fold)**
	**Alone**	**In mixture**		**Alone**	**In mixture**	
*Released HIV-1 virions from reactivated HIV-1 latent cells*						
T20	1.58	0.49	3.2	0.66	0.11	6.0
T2635	1.58	0.35	4.5	0.66	0.14	4.7
SFT	1.58	0.27	5.9	0.66	0.15	4.4

Data are representative of testing in triplicate (mean).
